# Genetic analysis of digital image derived morphometric traits of black tiger shrimp (*Penaeus monodon*) by incorporating G × E investigations

**DOI:** 10.3389/fgene.2022.1007123

**Published:** 2022-10-18

**Authors:** Md. Mehedi Hasan, Peter C. Thomson, Herman W. Raadsma, Mehar S. Khatkar

**Affiliations:** ^1^ The University of Sydney, Faculty of Science, Sydney School of Veterinary Science, Camden, NSW, Australia; ^2^ ARC Research Hub for Advanced Prawn Breeding, Townsville, QLD, Australia

**Keywords:** black tiger shrimp, breeding, morphological size, shape, heritability, genetic correlation, genotype-by-environment interaction

## Abstract

The black tiger shrimp, *Penaeus monodon*, is the second most economically important aquaculture shrimp species in the world, and in Australia it is one of the most commonly farmed shrimp species. Despite its economic significance, very few studies have reported the genetic evaluation of economically important morphological size and shape traits of shrimp grown in commercial grow-out environments. In this study we obtained genetic parameter estimates and evaluated genotype-by-environment interaction (GxE) for nine body morphological traits of shrimp derived from images. The data set contained image and body weight (BW) records of 5,308 shrimp, from 64 sires and 54 dams, reared in eight grow-out ponds for an average of 133 days. From the images, landmark based morphological distances were computed from which novel morphological traits associated with size and shape were derived for genetic evaluation. These traits included body weight (BW), body length (BL), body size (BS), head size (HS), Abdominal size (AS), abdominal percentage (AP), tail tip (TT), front by back ratio (FBR), condition factor (CF) and condition factor length (CFL). We also evaluated G×E interaction effects of these traits for shrimp reared in different ponds. The heritability estimates for growth related morphological and body weight traits were moderately high (BW: *h* ^2^ = *0*.32 ± 0.05; BL: *h* ^2^ = *0*.36 ± 0.06; BS: *h* ^2^ = *0*.32 ± 0.05; HS: *h* ^2^ = *0*.31 ± 0.05; AS: *h* ^2^ = *0*.32 ± 0.05; and TT: *h* ^2^ = *0*.28 ± 0.05) and low for abdominal percentage and body shape traits (AP: *h* ^2^ = *0*.09 ± 0.02; FBR: *h* ^2^ = *0*.08 ± 0.02; CF: *h* ^2^ = *0*.06 ± 0.02; and CFL: *h* ^2^ = *0*.003 ± 0.004). G × E interaction were negligible for all traits for shrimp reared in different ponds, suggesting re-ranking is not prevalent for this population. Genetic correlations among growth related morphological traits were high ranging from 0.36 to 0.99, suggesting these traits can be simultaneously improved through indirect genetic selection. However, negative genetic correlations were observed for FBR & CF shape traits with major growth traits (ranged −0.08 to −0.95), suggesting genetic selection for rapid growth will likely result in thick/fatty shrimp over generations. Our study showed image-based landmark data can be successfully employed for genetic evaluation of complex morphological traits of shrimp and is potentially amenable to machine-learning derived parameters in semi-automated high volume phenotyping systems needed under commercial conditions.

## Introduction

The black tiger shrimp (*Penaeus monodon*), hereafter shrimp, is one of the most commercially important aquaculture species in the world (Van Sang et al., 2020), and is the most popular cultivated shrimp species in Australia (APFA, 2016). However, most of the local demand is met by imports from overseas (FRDC, 2020). Due to this economic burden, and the significant biosecurity risks to Australia from imported products, there is a need for improvement of shrimp aquaculture in Australia. Traditionally, Australian shrimp aquaculture is dependent on the collection of wild broodstock for generating seedstock for stocking in aquaculture farms. However, a comparative study between domesticated and wild Australian shrimp populations revealed a 39% higher yield in the selectively bred stocks, suggesting a great potential of genetic improvement of this species in Australia (Norman-Lόpez et al., 2016). To address this, the application of genetic selection in shrimp will increase the genetic merit of locally bred stocks and farm profitability.

The most economically important traits in most shrimp breeding programs are body size and body weight, which are a measure of growth, and shape of the animal. Most aquaculture selective breeding programs focus on the genetic improvement of growth traits (e.g., weight and size), as it ensures higher yields. However, shape traits (e.g., key body conformation ratios, head size, abdominal size etc.) are equally important as candidate traits for genetic improvement (Cardoso et al., 2021). Shrimp that have more depth and width tend to yield more edible meat, compared to thinner and slender shaped ones. Moreover, animals which are uniform in size and shape are preferred by both industry and consumers. However, compared to finfish species, and due to difficulties in measuring shape and body shape index traits in shrimp, genetic evaluation of morphological traits in shrimp has not been performed. For example, due to natural curvature seen in the body structure of shrimp, it is difficult to compute body shape indexes, such as the condition factors. It has been demonstrated that landmark-based morphometric distance data derived from shrimp digital images can be used to evaluate and define complex body size and shape traits, such as body size and various aspects of morphological ratios (Hasan et al., 2020). This approach is robust and simple to assess complex traits, which are generally difficult to measure for shrimp, and therefore creates a novel opportunity to evaluate genetic parameters of these traits in shrimp.

An important aspect of all aquaculture production is to understand the underlying biological processes involved in size and shape of the animal, as it is reflected in the final product quality found in the marketplace. A key component of this is the estimation of genetic parameters associated with size and shape traits. Genetic improvement in shrimp aquaculture production can be made by developing selection programs utilizing these genetic parameter estimates. In Australia, genetic parameter estimates for growth traits have been previously studied for the black tiger shrimp in the tank environments (Kenway et al., 2006; Coman et al., 2010; Sun et al., 2015). However, no studies have been carried out so far in the pond environment, which is the usual commercial grow-out environment for most of the aquaculture production farms. It is very important to know the heritability estimates in pond environments rather than in the tanks, otherwise the estimates could be biased or confounded.

Furthermore, as with other aquaculture breeding programs, shrimp are often selected in one breeding station and are then distributed across different grow-out ponds or environments. To exploit the full genetic potential in the breeding program of a species, it is important to understand the performance and extent of realized genetic gains across environments. Genotype-by-environment interaction (G × E), which is the variable expression of phenotypes of genetically identical organisms in different environments, can occur if genotypes are not well adapted in their respective rearing environments. G × E reduces the efficiency of selection breeding programs, since the best-performing genotypes in one environment are not necessarily the best in another environment. Meta-analysis studies with both fish and shrimp species have reported the presence of significant G × E for growth traits (Sae-Lim et al., 2016; Hasan et al., 2020).

The present study was conducted to provide genetic parameter estimates of novel digital image-derived size and shape traits of shrimp from an Australian population of this species. In addition to this, we investigated the extent of G × E interactions in the shrimp population studied, which were raised across different grow-out ponds.

## Material and methods

### Family production, grow-out, pedigree construction and phenotype acquisition

All progeny in this experiment were sampled from commercial cohorts of *P. monodon* raised by Seafarms Group Ltd., as described in Foote et al. (2019). Briefly, wild broodstock were sourced from Joseph Bonaparte Gulf, Northern Territory, Australia and transferred to a commercial hatchery at Flying Fish Point, Queensland, Australia. Broodstock maturation was conducted within indoor flow-through tank systems (density of 3 m^−2^ at 28 ± 0.5°C) and broodstock were fed a commercial maturation diet. For each cohort, broodstock were allowed to mate naturally within the tanks, with any unmated females then artificially inseminated following industry practices. Females were spawned in communal spawning tanks and spawned eggs were transferred hatching tanks, and hatched nauplii were then transferred into 20,000-L larval rearing tanks (LRTs) at a density from 100 to 125 nauplii/L, and reared on a commercial diet (Ridley Aqua Feed, Australia) until 30 days of culture (DOC). LTRs were then pooled and stocked into seven 4000-m^2^ grow-out earthen ponds and reared under commercial conditions at a density of 45 m^−2^ until harvest. Preharvest ponds were immediately sampled by random cast net. From post-larval stage 15 (PL15) to harvest, the growth periods ranged from 124 to 143 days across ponds. In total 76 full sib and half-sib families were produced across 5,308 progeny and stocked across eight ponds as shown in Supplementary Material S1 and was similar to the population described by Noble et al. (2020b). Through the grow-out period, the key water quality parameters were recorded, including dissolved oxygen, temperature, pH and salinity.

For the genetic evaluation, 5,308 shrimp that were weighed and photographed, were included. Twelve landmarks’ data points were manually captured from the photographed images of shrimp as described in Hasan et al. (2019). These consist of one anterior, two posterior, four dorsal and five ventral landmarks. From these landmark data points, 66 morphological distance measurements were derived from the Euclidean distances between all pairwise coordinates. A principal component analysis (PCA) using the “prcomp” function in R was performed to describe the pattern of shrimp shape variation using these 66 morphological distances. From the component loadings of the PCA, key traits associated with shrimp morphology were defined as 1) body length (BL), from the most anterior point of the antennal scale to most posterior point of the tail based on the first principal component (PC1), 2) front to back ratio (FBR) between front body area (head and first abdominal segment) and the back area (abdominal segment two, three and four) based on PC2, and 3) condition factor (CF) calculated as (BW/BL^3^) × 100, where BW = body weight, BL = body length, based partly on PC3. Moreover, additional important morphological traits were obtained from the landmark-derived morphological distance data, e.g. body size (BS), head size (HS) and abdominal size (AS) by summing appropriate triangular areas using Heron’s formula. In addition, abdominal percentage (AP) was calculated as (AS/BS) × 100, tail tip (TT), the dorso-ventral distance across the tail, and condition factor length (CFL) calculated as (BS/BL^2^) × 100; where BS = body size, BL = body length.

Since tracing of broodstock contribution could not be done on farm, all potential broodstock were genotyped and parentage analysis was utilized to determine the contributing parents retrospectively as detailed by Guppy et al. (2020) and Noble et al. (2020a). A genotype-by-sequencing (GBS)-based approach using DArTSeq (Sansaloni et al., 2011) for genotyping of single nucleotide polymorphisms (SNPs) was used for the broodstock. This DArTSeq data set was used to derive a targeted 4 K DArTcap custom SNP panel of 4,194 SNPs for genotyping of the offspring (Guppy et al., 2020). CERVUS version 3.0.7 (Kalinowski et al., 2007) was used to perform family assignment, and Colony V2.0.6.4 (Jones and Wang, 2010) was employed to cluster the offspring to the genetic group when the parental information was missing, in which case an arbitrary parental ID was given to each group.

The family diversity of shrimp within each pond, which is a measure of the evenness of the distribution of families within ponds, was estimated by calculating the Shannon-Wiener Index using the “vegan” package in “R” (Oksanen et al., 2020). The index is calculated as

$$H=-\overset{n}{\underset{i=1}{\sum }}p_{i}\log_{e}p_{i}$$

where *p*_*i*_ is the proportion of animals in the pond from family *i*; maximum diversity occurs when families are represented equally (all *p*_*i*_ equal) resulting in *H*_max_ = –log_*e*_ *n*. The proportional family overlaps between pairs of ponds were calculated by the Morisita-Horn index, employed in the “divo” package in “R” (Sadee et al., 2019). The index for a pair of ponds (1,2) is calculated as

$$MH=2\overset{n}{\underset{i=1}{\sum }}p_{i1}p_{i2}/\left(\overset{n}{\underset{i=1}{\sum }}p_{i1}^{2}+\overset{n}{\underset{i=1}{\sum }}p_{i2}^{2}\right)$$

where *p*_*i*1_ and *p*_*i*2_ are the proportion of shrimp from family *i*, in ponds 1 and 2, and ranges from 0 (no overlap: entirely different families) to 1 (identical family representation).

### Statistical analysis

Initial exploratory analysis of the data was carried out by inspecting the distribution and homogeneity of variance assessed by performing Shapiro-Wilk and Levene’s tests. An ANOVA was performed to evaluate the significance of fixed effect of ponds. Quantitative genetic analysis such as (co)variance components, heritabilities (*h* ^2^), and genetic correlations (*r*_*g*_) between traits were estimated by restricted maximum-likelihood (REML) methods in ASReml-R 4.0 (VSNi) (Butler et al., 2017) by fitting the following animal model:

y=Xβ+Za+e

where **y** is the vector of observations of each trait (namely, BW, BL, BS, HS, AS, AP, TT, CF, CFL, TT, and FBR), **β** is the vector of fixed effects (“pond”, eight levels)*,* **a** is the vector of the random animal additive genetic effects, and **e** is the vector of random residual effects. Further, **X** and **Z** are corresponding incidence matrices for **β** and **a**, respectively.

Heritability was estimated from the following equation: $h^{2}=\sigma_{A}^{2}/\left(\sigma_{A}^{2}+\sigma_{e}^{2}\right)$ , where $\sigma_{A}^{2}$ = variance due to additive genetic components and $\sigma_{e}^{2}$ = variance arising from residual effects, respectively. A series of bivariate models were fitted to estimate covariance components among different traits in order to estimate genetic (*r*_*g*_) phenotypic (*r*_*p*_) and environmental (*r*_*e*_) correlations. Bivariate animal models were also used to estimate genotype by environment interaction (G×E). Here, G×E was estimated by calculation of covariance components of the same trait of shrimp in different ponds (Falconer, 1952). Genetic correlations (*r*_*g*_) were calculated from the following equation: $r_{g}=\sigma_{A12}/\sqrt{\sigma_{A1}^{2}\times \sigma_{A2}^{2}}$ , where $\sigma_{A1}^{2}$ and $\sigma_{A2}^{2}$ are the estimated additive genetic variance components of the same trait in different ponds (labelled 1, 2) and $\sigma_{A12}$ is the estimated genetic covariance between the pair of ponds.

Indirect genetic selection was calculated as a correlated response in trait *y* with 1 SD selection differential in trait *x* from the following equation:

$$CR_{y}=r_{g}\times h_{x}\times h_{y}\times SD_{y}$$

where *SD*_*y*_ is the SD of trait *y*.

The correlated response in trait *y* as a percentage of gain possible from direct selection for trait *x* is calculated as %IS, the relative efficiency of correlated response in trait *y* when selection is applied on trait *x* as a percentage of gain possible from direct selection for trait *y*, i.e.,

$$\%\text{IS}=\frac{CR_{y}}{SD_{y}}\times 100=\left(r_{g}\times h_{x}/h_{y}\right)\times 100.$$

## Results

### Family genetic diversity and descriptive statistics

The population studied was comprised of 5,308 individual F_1_ shrimp produced from 64 sires and 54 dams. These individual shrimp were raised in seven commercial ponds. The analysis of Shannon-Wiener diversity index revealed that families were not evenly distributed across ponds (Table 1). For example, a few families were over-represented in some ponds, particularly Pond 161 which had the lowest diversity for both sire families (*H* = 2.25) and dam families (*H* = 2.15), with similar low diversity in Pond 160. In contrast, Pond 155 had the most even distribution of families (sire families: *H* = 3.49; dam families: *H* = 3.17). Family distributions across ponds were also assessed by calculating the Morisita-Horn index (Table 2). It measures the family similarity across ponds and ranges from zero (no overlap) to one (perfect overlap). Here, Ponds 160 and 161 showed a very high Morisita-Horn overlap index between them (*MH* = 0.97), the same ponds with low family diversity, but low overlap with all other ponds suggesting unequal family representation across ponds. Supplementary Material S1 shows the distribution of the 76 families across the eight ponds.

**TABLE 1:** Shannon-Wiener diversity index of prawn families across ponds.

Pond	149	150	152	155	156	157	160	161
Sire	3.35	3.39	2.42	3.49	3.23	2.45	2.33	2.25
Dam	3.06	3.10	2.32	3.17	3.00	2.28	2.20	2.15

**TABLE 2:** Morisita-Horn overlap (similarity) index of prawn family distribution across ponds (a) sire (below diagonal) and (b) dam (above diagonal).

Pond	**149**	**150**	**152**	**155**	**156**	**157**	**160**	**161**
**149**		0.96	0.56	0.90	0.96	0.52	0.15	0.16
**150**	0.95		0.54	0.94	0.94	0.51	0.16	0.16
**152**	0.56	0.53		0.39	0.55	0.95	0.02	0.03
**155**	0.89	0.93	0.39		0.87	0.37	0.19	0.18
**156**	0.95	0.92	0.55	0.84		0.50	0.17	0.18
**157**	0.52	0.50	0.95	0.37	0.50		0.18	0.17
**160**	0.10	0.10	0.02	0.19	0.11	0.17		0.97
**161**	0.11	0.10	0.03	0.18	0.13	0.16	0.97	

Mean, standard deviation (SD) and coefficient of variation (CV) of the traits studied are shown in Table 3. Shrimp had an average weight of 14.5 g, average body length of 10.8 cm, and abdominal percentage of 63.8% (Table 3). Body weight (BW) was the most variable trait (CV of 27%) while Abdominal percentage (AP) was the least variable (2.3%).

**TABLE 3:** Mean, standard deviation (SD), coefficient of variation (CV %), additive genetic variance ( $\sigma_{A}^{2}$ ), residual variance component ( $\sigma_{e}^{2}$ ) and estimated heritability (*h* ^2^) of morphological traits of shrimp (*n* = 5,308).

Trait	Mean	SD	CV (%)	$\sigma_{A}^{2}$	$\sigma_{e}^{2}$	*h* ^2^
Body weight (g) (BW)	14.52	3.92	27.0	4.56 ± 0.92	9.38 ± 0.52	0.32 ± 0.05
Body length (cm) (BL)	10.82	1.01	9.3	0.35 ± 0.07	0.61 ± 0.03	0.36 ± 0.06
Body size (cm^2^) (BS)	13.39	2.61	19.5	2.08 ± 0.42	4.27 ± 0.23	0.32 ± 0.05
Head size (cm^2^) (HS)	4.83	0.92	19.2	0.25 ± 0.05	0.56 ± 0.02	0.31 ± 0.05
Abdominal size (cm^2^) (AS)	8.56	1.71	20.0	0.87 ± 0.17	1.80 ± 0.10	0.32 ± 0.05
Abdominal percentage (%) (AP)	63.87	1.48	2.3	0.15 ± 0.03	1.48 ± 0.03	0.09 ± 0.02
Tail tip (TT)	1.17	0.13	11.9	0.004 ± 0.001	0.01 ± 0.0005	0.28 ± 0.05
Front by back ratio (FBR)	0.84	0.06	7.2	0.0001 ± 0.00005	0.002 ± 0.00005	0.08 ± 0.02
Condition factor (CF)	1.11	0.07	6.6	0.0003 ± 0.0001	0.005 ± 0.0001	0.06 ± 0.02
Condition factor length (CFL)	11.30	0.52	4.6	0.0008 ± 0.001	0.25 ± 0.005	0.003 ± 0.004

### Heritability estimates

**FIGURE 1:**
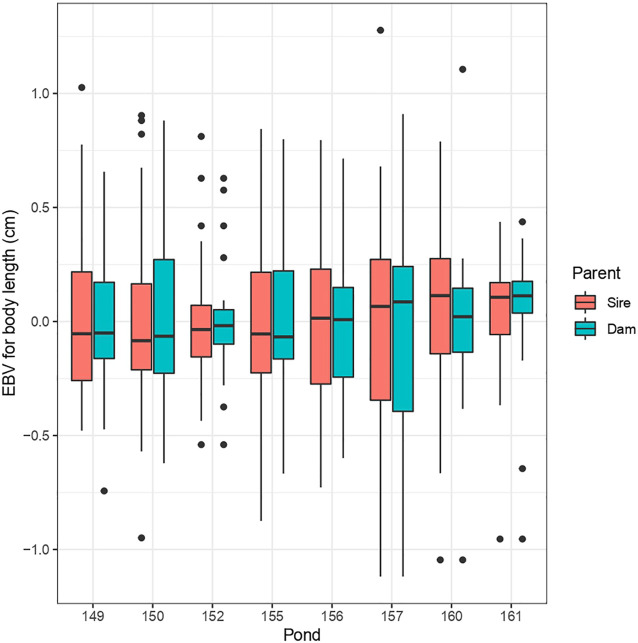
Distribution of EBVs for body length (BL) across ponds for sires and dams.

Heritability (*h* ^2^) estimates were moderate for BW (0.32 ± 0.05), BL (0.36 ± 0.06), BS (0.32 ± 0.05), HS (0.31 ± 0.05) and AS (0.32 ± 0.05) and were low for AP (0.09 ± 0.02), FBR (0.08 ± 0.02), CF (0.08 ± 0.02) and CFL (0.003 ± 0.004). With the exception of CFL, all estimates were significantly greater than zero based on their small standard errors (SE) (0.004–0.06). However, for CFL the *h* ^*2*^ estimate was near zero, indicating presence of minimal additive genetic variability in this trait for selection. To evaluate the extent of family performance across ponds, estimated breeding values (EBVs) of sires and dams were obtained for each pond, and an illustration of this is shown for body length in Figure 1. This considerable variability of EBVs is consistent with the estimated genetic SD for this trait (−0.6 cm), and this indicates there is potential for genetic improvement through selective breeding.

### Genetic correlations

Genetic correlations (*r*_*g*_) among BW, BL, BS, HS, AS and TT were high (range: *r*_*g*_ = 0.96–0.99) (Table 4). The *r*_*g*_ was medium with AP trait with the abovementioned traits (range: *r*_*g*_ = 0.36–0.40), but highly negative with FBR (*r*_*g*_ = -0.96). The phenotypic and environmental correlations were in similar alignment in both direction and magnitude as the genetic correlations for relationships between traits (Table 4).

**TABLE 4:** Genetic (*r*_*g*_ ± se), phenotypic (*r*_*p*_ ± se), and environmental (*r*_*e*_ ± se) correlations among traits and extent of indirect selection response in correlated traits.

Trait *x*	Trait *y*	*r*_*g*_ ± se	*r*_*p*_ ± se	*r*_*e*_ ± se	^*^CR_*y*_	% IS
BW^#^	BL	0.99 ± 0.002	0.95 ± 0.001	0.94 ± 0.003	0.34	105
BW	BS	0.99 ± 0.001	0.95 ± 0.001	0.93 ± 0.003	0.32	99
BW	HS	0.99 ± 0.002	0.93 ± 0.002	0.90 ± 0.005	0.31	101
BW	AS	0.99 ± 0.002	0.93 ± 0.001	0.95 ± 0.003	0.32	99
BW	AP	0.36 ± 0.14	0.17 ± 0.01	0.13 ± 0.02	0.06	19
BW	TT	0.96 ± 0.01	0.86 ± 0.005	0.82 ± 0.009	0.29	103
BW	FBR	−0.48 ± 0.13	−0.19 ± 0.01	−0.15 ± 0.02	−0.08	−96
BW	CF	−0.63 ± 0.13	0.05 ± 0.01	0.16 ± 0.02	−0.09	−145
BL	BS	0.99 ± 0.0006	0.96 ± 0.001	0.95 ± 0.002	0.34	105
BL	HS	0.99 ± 0.001	0.94 ± 0.002	0.92 ± 0.004	0.33	107
BL	AS	0.99 ± 0.001	0.96 ± 0.001	0.95 ± 0.002	0.34	105
BL	AP	0.36 ± 0.14	0.15 ± 0.02	0.18 ± 0.01	0.06	72
BL	TT	0.97 ± 0.008	0.89 ± 0.004	0.85 ± 0.007	0.31	110
BL	FBR	−0.49 ± 0.13	−0.21 ± 0.01	−0.16 ± 0.02	−0.08	−102
BL	CF	−0.69 ± 0.12	−0.17 ± 0.01	0.11 ± 0.02	−0.1	−169
BS	HS	0.99 ± 0.001	0.98 ± 0.0007	0.97 ± 0.001	0.32	99
BS	AS	0.99 ± 0.0003	0.99 ± 0.0002	0.99 ± 0.0004	0.32	99
BS	AP	0.40 ± 0.14	0.15 ± 0.01	0.10 ± 0.02	0.07	75
BS	TT	0.97 ± 0.007	0.92 ± 0.002	0.90 ± 0.004	0.29	104
BS	FBR	−0.53 ± 0.12	−0.17 ± 0.01	−0.11 ± 0.02	−0.08	−106
BS	CF	−0.67 ± 0.12	−0.11 ± 0.01	−0.03 ± 0.02	−0.09	−155
HS	AS	0.99 ± 0.003	0.95 ± 0.001	0.94 ± 0.003	0.31	97
HS	TT	0.97 ± 0.009	0.89 ± 0.004	0.85 ± 0.007	0.29	102
HS	FBR	−0.45 ± 0.14	−0.02 ± 0.01	0.06 ± 0.02	−0.07	−89
HS	CF	−0.69 ± 0.12	−0.11 ± 0.01	−0.03 ± 0.02	−0.09	−157
AS	TT	0.97 ± 0.007	0.93 ± 0.002	0.91 ± 0.004	0.29	104
AS	FBR	−0.56 ± 0.12	−0.26 ± 0.01	−0.21 ± 0.02	−0.09	−112
AS	CF	−0.65 ± 0.12	−0.11 ± 0.01	0.03 ± 0.02	−0.09	−150
AP	FBR	−0.96 ± 0.01	−0.88 ± 0.003	−0.87 ± 0.003	−0.16	−51
AP	CF	−0.08 ± 0.20	0.001 ± 0.01	0.008 ± 0.01	−0.01	−10
TT	FBR	−0.57 ± 0.12	−0.30 ± 0.01	−0.26 ± 0.02	−0.09	−30
TT	CF	−0.67 ± 0.12	−0.12 ± 0.01	−0.04 ± 0.02	−0.09	−31
TT	CFL	0.81 ± 0.31	0.40 ± 0.01	0.45 ± 0.01	0.02	8
FBR	CF	0.20 ± 0.20	0.003 ± 0.01	−0.01 ± 0.01	0.01	23
FBR	CFL	−0.96 ± 0.29	0.04 ± 0.01	0.08 ± 0.01	−0.01	−19
CF	CFL	0.28 ± 0.42	0.29 ± 0.03	0.29 ± 0.04	0.00	125

*Correlated response in trait *y* with 1 SD, selection differential in trait *x*. %IS, relative efficiency of correlated response in trait *y* when selection is applied on trait *x* as a percentage of gain possible from direct selection for trait *y.*

^#^BW, body weight (g); BL, body length; BS, body size; HS, head size; AS, abdominal size; AP, abdominal percentage; TT, tail tip; FBR, front-back ratio; CF, condition factor; CFL, condition factor length. Note that missing trait pairs correspond to when the bivariate model could not be fitted.

### G × E across ponds

**FIGURE 2:**
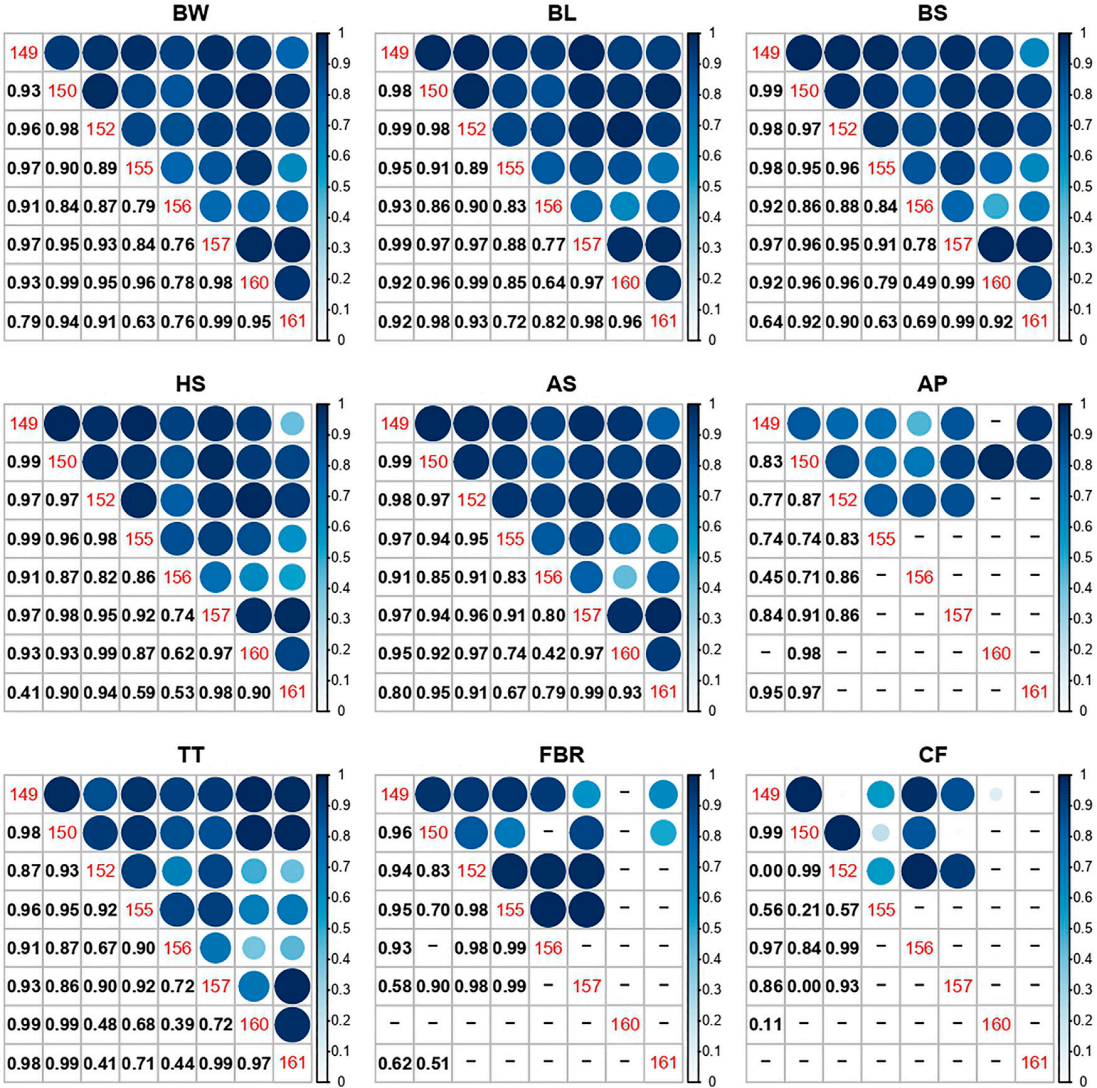
Correlation plots of estimated genetic correlations of traits between pairs of ponds, as a measure of G × E. Pond numbers are shown down the diagonal, with numerical values below the diagonal, and correlation visualisation above. Values shown as “–” are where the particular bivariate model could not be fitted. Trait codes are BW = body weight (g); BL = body length; BS = body size; HS = head size; AS = abdomenal size; AP = abdominal percentage; TT = tail tip; FBR = front-back ratio; CF = condition factor. Condition factor length (CFL) not shown as only one correlation could be estimated (Ponds 149 and 152: *r*_*g*_ = 0.99).

The genetic correlation between phenotypic expressions of the same traits in different ponds indicated lack of any G × E effect for all the traits studied (Figure 2, Supplementary Material S2). Although, there were indications of G × E among certain ponds (e.g., for BS trait between pond 155 & 161, 0.63 ± 0.45). However, the very high SE in these instances reflects the limited overlap of families in these pairs of ponds, limiting the ability to infer G × E or otherwise, for these ponds.

## Discussion

After *Penaeus vannamei*, *Penaeus monodon* is the next most important cultivated shrimp species in the world. However, compared to its aquaculture importance, genetic improvement of this species has been quite limited. Although some previous genetic parameter estimates have been reported for this species, they were all derived in tank environments. In commercial breeding programs it is important to simulate commercial conditions during genetic evaluation, so that the animals evaluated and selected for best targeted traits in the breeding nucleus population demonstrate similar yields in commercial settings. In this study, the performance of shrimp was evaluated in standard Australian farming conditions.

Growth traits are often considered the most important traits in any breeding programs due to their direct correlation with the economic value of the product. Overall, moderate to high heritability estimates were recorded for BW (0.32 ± 0.05), BL (0.36 ± 0.06) and BS (0.32 ± 0.05), suggesting substantial potential for genetic improvement of these traits in the *P. monodon* population studied. All these are growth-related traits and these findings corroborate the previous findings of *P. monodon* genetic parameter estimates (Kenway et al., 2006; Macbeth et al., 2007; Coman et al., 2010; Noble et al., 2020b), although a meta-analysis conducted by Hasan et al. (2020) reported the estimates ranged from 0.18 to 0.69. Similarly Noble et al. (2020b) reported the heritability estimate of 0.38 for BW trait of *P. monodon* for a smaller subset of this population. A higher heritability of 0.55 for growth rate (at 54 weeks) and 0.47 body weight were reported by Kenway et al. (2006) and Van Sang et al. (2020), respectively. However, Sun et al. (2015) reported low heritability estimates for body length (0.18) and body weight (0.24) trait of this species. Similarly, Krishna et al. (2011) also reported a low heritability estimate of 0.27 for body weight trait of *P. monodon*. There could be various factors that could lead to high level of heterogeneity of heritability estimates of any traits, including common family environmental effects (Rutten et al., 2005), characteristics of the grow-out farm (Turra et al., 2012), sex and age of the animal (Benzie et al., 1997) etc. For example, for body length, Benzie et al. (1997) reported a heritability of 0.59 at 6 weeks and 0.30 at 10 weeks of age for *P. monodon*.

In addition to these growth traits, heritability of traits related to shape e.g., HS (0.31 ± 0.05) and AS (0.32 ± 0.05) were moderately high, and low for AP (0.09 ± 0.02), FBR (0.08 ± 0.02), CF (0.06 ± 0.02) and CFL (0.003 ± 0.004) traits. This suggests that HS and AS traits are suitable for selection to change body shape of shrimp. Shrimp with smaller HS, larger AS and higher AP might be preferable for selection, as it may correspond directly to higher meat yield. Moreover, to attain uniformity of the cultured shrimp body shape, FBR trait can be selected in combination with these other traits by employing multi-trait selection criteria. Until now, no genetic analysis has been conducted on shape traits of shrimp. Quantitative trait loci (QTL) and genome wide association studies (GWAS) with Gilthead seabream (*Sparus aurata*), sea bass (*Dicentrarchus labrax*) and common carp (*Cyprinus carpio*) have shown that economically important shape-related traits are associated with unique genomic regions (Colihueque and Araneda, 2014). However, to date no specific study has been undertaken to search for genetic associations in shrimp for shape traits. Recently, due to increasing market sophistication, shape traits are gaining special attention by aquaculture breeders and consumers (Mehar et al., 2020). For example, high-backed and elliptical shaped common carp have high economic value and are commercially cultivated in fish farms. In the case of the ornamental goldfish (*Carassius auratus*), various morphological traits (e.g., body shape, fin morphology, and eye features) are modified *via* selective breeding to increase economic value (Colihueque and Araneda, 2014). Similarly, body shape traits of black tiger shrimp can be selected according to economic value and consumer preferences, e.g., shrimp with smaller heads may be preferred to increase meat yield, or shrimp with uniform size and shape may also be preferred by retailers and consumers (Mehar et al., 2020). This can be achieved through considering the genetic correlations of growth and morphological shape traits in the selective breeding program.

In our current study, genetic correlations between key growth-related morphological traits were positive and high (e.g., between and among BW, BL, BS, AS, and HS). This suggests that key traits for growth and shape of *P. monodon* can be selected indirectly. Our estimates of correlated responses among different traits indicates that selection on BS trait can indirectly improve BW and AP traits by 99% and 75%, respectively. This indicates these traits are a likely to be regulated by some of the same genes. A similar trend was observed among other morphological traits. Thus, selection of one such morphological trait will lead to change in other correlated morphological traits. In the case of rainbow trout, selection for increased BW indirectly resulted in fish with higher body width and height with rounded shape (Kause et al., 2003). However, a negative association was seen between FBR and CF traits with all other morphological traits, suggesting a potential trade-off is required in selecting shrimp body shape traits. This trade-off indicates the presence of certain limits in the selection of FBR (shape) trait, that is direct selection on these morphological traits will lead to decrease in FBR in this shrimp population, i.e., there will be thicker shaped shrimp. It is well known that functional interdependence among various traits plays a key role in the constraining the evolution of a certain shape. For example, there has been a report on the relationship between body shape and physical activity in fish (e.g., swimming performance) (Reid and Peichel, 2010). A similar underlying phenomenon may be responsible for the CF trade-off for shrimp, which warrants further investigation.

Genetic correlation estimates (as a measure of G×E) between ponds with high Morisita-Horn index scores (>0.56) were high for BW, BL, BS and HS. Only moderately low G×E (*r*_*g*_ = 0.74 ± 0.29 and 0.45 ± 0.30) was observed in AP, for pond pairs of 149 and 155 and 149 and 156, respectively. This suggests that G × E interactions were non-existent for these traits and re-ranking of breeding values may be less pronounced across the ponds in this shrimp population, but this may be expected given all the ponds are in the one location. Similar to this finding, a meta-analysis, using data from 29 peer reviewed studies, found that growth-related morphological traits have a high genetic correlation (*r*_g_ = 0.73 ± 0.05) across various environments and species, indicating low levels of G × E with low re-ranking of breeding values across environments (Hasan et al., 2020). Being a non-fitness trait, growth-related morphological traits are less likely to be affected by variation in specific environments (Mousseau and Roff, 1987; Visscher et al., 2008). However, G × E for growth-related traits is evident in shrimp species when the grow-out environment is stressful (e.g., temperature, salinity, ammonia tolerance etc.) (Coman et al., 2002; Li et al., 2015). This indicates re-ranking can be present even for non-fitness traits (e.g., growth) when the environment is stressful.

For ponds with low family diversity (e.g., pond 160 and 161), low genetic correlation estimates were recorded for BW, BL, BS and HS. Although this could be an indication of high G × E, the estimates are unreliable due to their large standard error. This suggests the importance of maintaining homogenous family distribution across experimental grow-out environments, to obtain reliable G × E estimates.

For efficient improvement of traits under selection, the recording of these traits should be accurate and cost effective. Our study demonstrates that digital images can be used to derive economically-important growth-related morphological traits from landmark data to evaluate genetic merit. While in most breeding programs, body weight is the key trait for selection, it can be predicted from image-derived data as was the case in this study. So, if the breeding goal is to achieve a specific shape of the animal, and taking weight record is not feasible, then body measurements can be an excellent option to examine shrimp production traits. From our image-based analysis of morphological traits, we were able to derive size and shape measurements of the shrimp (e.g., length, width, abdominal percentage, abdominal size, FBR etc.) which can then be used for selection. For example, meat yield (peeled tail weight) of shrimp may not be feasible to record due to technical difficulties, however abdominal size can be used to infer the meat weight. Similarly, to select shrimp with a thick structure (FBR), body size (BS) trait can be selected. However, future studies are required for 1) phenotyping of morphological traits directly from the images using machine learning approaches without the need to do manual landmarking, and 2) obtaining estimates of economic values of the morphological traits, as these are unknown and need to be studied before incorporating in the breeding programs.

## Conclusion

Our study shows that landmark-based digital-image analysis is a promising tool of phenotyping of shrimp morphological traits and for genetic evaluation of these traits. Genetic improvement for growth-related morphological and weight traits is feasible, since these traits demonstrated high genetic variation and heritability. Family-level selection breeding is needed for genetic improvement of some shape traits (e.g., FBR), as the heritability was low. Within the same farm, it is not essential to perform separate genetic evaluations of shrimp across multiple ponds as G × E effects were found to be negligible between ponds.

## Data Availability

The original contributions presented in the study are included in the article/Supplementary Materials, further inquiries can be directed to the corresponding author.
