# Supplementary material for: Genetic analysis of digital image derived morphometric traits of black tiger shrimp (Penaeus monodon) by incorporating G × E investigations
Source: Front Genet. 2022 Oct 18;13:1007123. doi: 10.3389/fgene.2022.1007123 (PMC9632751; doi:10.3389/fgene.2022.1007123)
Supplement: Supplementary file 1 [file DataSheet2.PDF]

family	149	150	152	155	156	157	160	161	All_ponds
G1T1701_BR_002_G1T1701_BR_048	30	19		36	13				98
G1T1701_BR_002_G1T1701_BR_050	13	14		15	9				51
G1T1701_BR_003_G1T1701_BR_049	28	34	19	16	39				136
G1T1701_BR_004_G1T1701_BR_052				1					1
G1T1701_BR_005_G1T1701_BR_053	25	16	7	17	13				78
G1T1701_BR_006_G1T1701_BR_053	31	23	20	53	31				158
G1T1701_BR_007_G1T1701_BR_052	1	1	2	2					6
G1T1701_BR_008_G1T1701_BR_$4	37	46	192	24	33	181			513
G1T1701_BR_010_G1T1701_BR_$7						17	21	13	51
G1T1701_BR_010_G1T1701_BR_055		1		1					2
G1T1701_BR_012_G1T1701_BR_057	1	9		15	2				27
G1T1701_BR_013_G1T1701_BR_055		1							1
G1T1701_BR_014_G1T1701_BR_057	7	6		10	4				27
G1T1701_BR_015_G1T1701_BR_119	9	8		11	2				30
G1T1701_BR_017_G1T1701_BR_059						2			2
G1T1701_BR_017_G1T1701_BR_068	13	5	6	5	8				37
G1T1701_BR_018_G1T1701_BR_059			3			5			8
G1T1701_BR_023_G1T1701_BR_062	60	43	26	28	83	15	20	31	306
G1T1701_BR_024_G1T1701_BR_062	25	20		31	14	1	1	1	93
G1T1701_BR_030_G1T1701_BR_$1	7	4	37	4	8	49			109
G1T1701_BR_031_G1T1701_BR_064	7	1	7	3	13	1			32
G1T1701_BR_031_G1T1701_BR_168								1	1
G1T1701_BR_033_G1T1701_BR_064	9	10	3	6	4				32
G1T1701_BR_035_G1T1701_BR_065						13	9	16	38
G1T1701_BR_036_G1T1701_BR_065			3			11	5	4	23
G1T1701_BR_037_G1T1701_BR_067	23	39	12	33	39				146
G1T1701_BR_038_G1T1701_BR_067	16	21	3	13	22				75
G1T1701_BR_041_G1T1701_BR_170	4	5			2				11
G1T1701_BR_041_G1T1701_BR_461	4	4	4	5	8				25
G1T1701_BR_042_G1T1701_BR_139	42	31	19	25	46				163
G1T1701_BR_044_G1T1701_BR_198						7	12	9	28
G1T1701_BR_141_G1T1701_BR_192			2			23	55	46	126
G1T1701_BR_251_G1T1701_BR_123	3	4		10	4				21
G1T1701_BR_262_G1T1701_BR_113	19	21	6	20	19				85
G1T1701_BR_262_G1T1701_BR_131	123	100	129	71	122	125			670
G1T1701_BR_263_G1T1701_BR_166	26	37	6	35	53				157
G1T1701_BR_268_G1T1701_BR_403						1			1
G1T1701_BR_272_G1T1701_BR_$5	14	27		24	10				75
G1T1701_BR_278_G1T1701_BR_163				1					1
G1T1701_BR_283_G1T1701_BR_$3	6	2	2	7	6				23
G1T1701_BR_283_G1T1701_BR_095	1	1	3	6	4				15
G1T1701_BR_284_G1T1701_BR_362						26	46	57	129
G1T1701_BR_293_G1T1701_BR_401	5	14		23	8				50
G1T1701_BR_318_G1T1701_BR_360	30	40		43	39	13	9	9	183
G1T1701_BR_322_G1T1701_BR_060	3	4		12	1				20
G1T1701_BR_324_G1T1701_BR_119	21	28		25	11				85
G1T1701_BR_325_G1T1701_BR_102	1			2					3

G1T1701_BR_325_G1T1701_BR_402							66	71	137
G1T1701_BR_327_G1T1701_BR_$2	4	5		6	4				19
G1T1701_BR_348_G1T1701_BR_117				1					1
G1T1701_BR_359_G1T1701_BR_135						13	14	11	38
G1T1701_BR_363_G1T1701_BR_222	2	1	11	2		10			26
G1T1701_BR_367_G1T1701_BR_385			2			5			7
G1T1701_BR_428_G1T1701_BR_$6			13			10			23
G1T1701_BR_428_G1T1701_BR_211	3	10		7	5	1	3		29
G1T1701_BR_441_G1T1701_BR_123	1			1					2
G1T1701_BR_441_G1T1701_BR_388								1	1
G1T1701_BR_444_G1T1701_BR_055	5	9		7	10				31
G1T1701_BR_444_G1T1701_BR_134	11	12	1	14	9				47
G1T1701_BR_445_G1T1701_BR_$3	5	5	1	5	4				20
G1T1701_BR_445_G1T1701_BR_095	15	5	4	10	14				48
G1T1701_BR_450_G1T1701_BR_401	2	4		8	1				15
G1T1701_BR_|10_G1T1701_BR_211	13	16		31	16	4	8	3	91
G1T1701_BR_|11_G1T1701_BR_386	8	10	4	7	16				45
G1T1701_BR_|12_G1T1701_BR_187			19			20			39
G1T1701_BR_|13_G1T1701_BR_173			13			17			30
G1T1701_BR_|14_G1T1701_BR_391							16	13	29
G1T1701_BR_|1_G1T1701_BR_239	22	24	1	16	6	1			70
G1T1701_BR_|2_G1T1701_BR_131	20	31	19	16	15	22			123
G1T1701_BR_|3_G1T1701_BR_048	30	48		39	19				136
G1T1701_BR_|4_G1T1701_BR_049	7	2		3	4				16
G1T1701_BR_|5_G1T1701_BR_113	10	7		8	5				30
G1T1701_BR_|6_G1T1701_BR_231	8	7		1	3				19
G1T1701_BR_|7_G1T1701_BR_171	11	20		14	11				56
G1T1701_BR_|8_G1T1701_BR_360	24	33	4	44	23	23	30	24	205
G1T1701_BR_|9_G1T1701_BR_231	6	5		4	8				23
Total	851	893	603	877	843	616	315	310	5308
