# Supplementary material for: Genetic analysis of digital image derived morphometric traits of black tiger shrimp (Penaeus monodon) by incorporating G × E investigations
Source: Front Genet. 2022 Oct 18;13:1007123. doi: 10.3389/fgene.2022.1007123 (PMC9632751; doi:10.3389/fgene.2022.1007123)
Supplement: Supplementary file 2 [file DataSheet1.PDF]

Genetic analysis of digital image derived morphometric traits of black tiger shrimp (*Penaeus monodon*) by incorporating G×E investigations

Md. Mehedi Hasan, Peter C. Thomson, Herman W. Raadsma and Mehar S. Khatkar

Supplementary Material

G×E of traits across ponds for each of the ten morphological traits, as estimated by the genetic correlation (r_g)

(a) Body weight (BW, (g) above diagonal and body length (BL, cm) below diagonal.

**Pond**	**149**	**150**	**152**	**155**	**156**	**157**	**160**	**161**
**149**		0.93 ± 0.03	0.96 ± 0.14	0.97 ± 0.05	0.91 ± 0.08	0.97 ± 0.05	0.93 ± 0.37	0.79 ± 0.14
**150**	0.98 ± 0.03		0.98 ± 0.05	0.90 ± 0.06	0.84 ± 0.09	0.95 ± 0.06	0.99 ± 0.17	0.94 ± 0.20
**152**	0.99 ± 0.07	0.98 ± 0.05		0.89 ± 0.12	0.87 ± 0.12	0.93 ± 0.07	0.95 ± 1.15	0.91 ± 0.80
**155**	0.95 ± 0.06	0.91 ± 0.06	0.89 ± 0.10		0.79 ± 0.10	0.84 ± 0.14	0.96 ± 0.13	0.63 ± 0.45
**156**	0.93 ± 0.05	0.86 ± 0.07	0.90 ± 0.10	0.83 ± 0.09		0.76 ± 0.22	0.78 ± 0.33	0.76 ± 0.42
**157**	0.99 ± 0.03	0.97 ± 0.05	0.97 ± 0.05	0.88 ± 0.11	0.77 ± 0.21		0.98 ± 0.19	0.99 ± 0.07
**160**	0.92 ± 0.24	0.96 ± 0.22	0.99 ± 0.90	0.85 ± 0.22	0.64 ± 0.40	0.97 ± 0.18		0.95 ± 0.05
**161**	0.92 ± 0.18	0.98 ± 0.13	0.93 ± 0.70	0.72 ± 0.33	0.82 ± 0.32	0.98 ± 0.11	0.96 ± 0.05	

(b) Body size (BS, cm^2) above diagonal and head size (HS, cm^2) below diagonal.

**Pond**	**149**	**150**	**152**	**155**	**156**	**157**	**160**	**161**
**149**		0.99 ± 0.03	0.98 ± 0.16	0.98 ± 0.06	0.92 ± 0.07	0.97 ± 0.07	0.92 ± 0.28	0.64 ± 0.54
**150**	0.99 ± 0.04		0.97 ± 0.08	0.95 ± 0.06	0.86 ± 0.08	0.96 ± 0.06	0.96 ± 0.16	0.92 ± 0.20
**152**	0.97 ± 0.25	0.97 ± 0.10		0.96 ± 0.08	0.88 ± 0.12	0.95 ± 0.06	0.96 ± 0.99	0.90 ± 0.78
**155**	0.99 ± 0.06	0.96 ± 0.06	0.98 ± 0.08		0.84 ± 0.09	0.91 ± 0.10	0.79 ± 0.28	0.63 ± 0.45
**156**	0.91 ± 0.08	0.87 ± 0.08	0.82 ± 0.15	0.86 ± 0.09		0.78 ± 0.21	0.49 ± 0.47	0.69 ± 0.46
**157**	0.97 ± 0.10	0.98 ± 0.04	0.95 ± 0.06	0.92 ± 0.10	0.74 ± 0.23		0.99 ± 0.17	0.99 ± 0.08
**160**	0.93 ± 0.30	0.93 ± 0.19	0.99 ± 0.70	0.87 ± 0.22	0.62 ± 0.42	0.97 ± 0.19		0.92 ± 0.09
**161**	0.41 ± 0.71	0.90 ± 0.22	0.94 ± 0.45	0.59 ± 0.48	0.53 ± 0.59	0.98 ± 0.09	0.90 ± 0.10	

(c) Abdomen size (AS, cm^2) above diagonal and abdominal pe4rcentahe (AP, (%) below diagonal.

Pond	149	150	152	155	156	157	160	161
**149**		0.99 ± 0.03	0.98 ± 0.12	0.97 ± 0.06	0.91 ± 0.07	0.97 ± 0.06	0.95 ± 0.25	0.80 ± 0.38
**150**	0.83 ± 0.20		0.97 ± 0.06	0.94 ± 0.06	0.85 ± 0.09	0.94 ± 0.07	0.92 ± 0.21	0.95 ± 0.18
**152**	0.77 ± 0.29	0.87 ± 0.20		0.95 ± 0.08	0.91 ± 0.11	0.96 ± 0.06	0.97 ± 1.05	0.91 ± 0.97
**155**	0.74 ± 0.18	0.74 ± 0.17	0.83 ± 0.49		0.83 ± 0.09	0.91 ± 0.10	0.74 ± 0.31	0.67 ± 0.42
**156**	0.45 ± 0.30	0.71 ± 0.20	0.86 ± 1.19	NA		0.80 ± 0.20	0.42 ± 0.49	0.79 ± 0.38
**157**	0.84 ± 0.24	0.91 ± 0.18	0.86 ± 0.37	NA	NA		0.97 ± 0.19	0.99 ± 0.08
**160**	NA	0.98 ± 1.33	NA	NA	NA	NA		0.93 ± 0.08
**161**	0.95 ± 0.32	0.97 ± 0.59	NA	NA	NA	NA	NA	

(d) Tail tip (TT, cm) above diagonal and front-back ratio (FBR) below diagonal.

Pond	149	150	152	155	156	157	160	161
**149**		0.98 ± 0.04	0.87 ± 0.15	0.96 ± 0.09	0.91 ± 0.07	0.93 ± 0.09	0.99 ± 0.16	0.98 ± 0.19
**150**	0.96 ± 0.03		0.93 ± 0.08	0.95 ± 0.08	0.87 ± 0.08	0.86 ± 0.13	0.99 ± 0.13	0.99 ± 0.14
**152**	0.94 ± 0.05	0.83 ± 0.20		0.92 ± 0.13	0.67 ± 0.28	0.90 ± 0.16	0.48 ± 1.25	0.41 ± 1.73
**155**	0.95 ± 0.03	0.70 ± 0.20	0.98 ± 0.02		0.90 ± 0.09	0.92 ± 0.13	0.68 ± 0.42	0.71 ± 0.44
**156**	0.93 ± 0.04	NA	0.98 ± 0.02	0.99 ± 0.13		0.72 ± 0.25	0.39 ± 0.56	0.44 ± 0.85
**157**	0.58 ± 0.38	0.90 ± 0.19	0.98 ± 0.17	0.99 ± 0.01	NA		0.72 ± 0.43	0.99 ± 0.12
**160**	NA	NA	NA	NA	NA	NA		0.97 ± 0.06
**161**	0.62 ± 0.91	0.51 ± 1.01	NA	NA	NA	NA	NA	

(e) Condition factor (CF) above diagonal and condition factor length (CFL) below diagonal.

Pond	149	150	152	155	156	157	160	161
**149**		0.99 ± 0.03	-0.02 ± 0.46	0.56 ±0.27	0.97 ± 0.02	0.86 ± 0.18	0.11 ± 1.24	NA
**150**	NA		0.99 ± 0.03	0.21 ± 0.75	0.84 ± 0.57	-0.13 ± 0.83	NA	NA
**152**	0.99 ± 0.18	NA		0.57 ± 0.67	0.99 ± 0.02	0.93 ± 0.09	NA	NA
**155**	NA	NA	NA		NA	NA	NA	NA
**156**	NA	NA	NA	NA		NA	NA	NA
**157**	NA	NA	NA	NA	NA		NA	NA
**160**	NA	NA	NA	NA	NA	NA		NA
**161**	NA	NA	NA	NA	NA	NA	NA	
